# Distinguishing pure histopathological growth patterns of colorectal liver metastases on CT using deep learning and radiomics: a pilot study

**DOI:** 10.1007/s10585-021-10119-6

**Published:** 2021-09-17

**Authors:** Martijn P. A. Starmans, Florian E. Buisman, Michel Renckens, François E. J. A. Willemssen, Sebastian R. van der Voort, Bas Groot Koerkamp, Dirk J. Grünhagen, Wiro J. Niessen, Peter B. Vermeulen, Cornelis Verhoef, Jacob J. Visser, Stefan Klein

**Affiliations:** 1 Department of Radiology and Nuclear Medicine, Erasmus MC, Rotterdam, The Netherlands; 2 Department of Surgery, Erasmus MC Cancer Institute, Rotterdam, The Netherlands; 3 Faculty of Applied Sciences, Delft University of Technology, Delft, The Netherlands; 4 Translational Cancer Research Unit, Department of Oncological Research, Oncology Center, GZA Hospitals Campus Sint-Augustinus and University of Antwerp, Antwerp, Belgium

**Keywords:** Liver neoplasms, Machine learning, Biomarkers, Tomography, X-ray, Computed, Deep learning

## Abstract

Histopathological growth patterns (HGPs) are independent prognosticators for colorectal liver metastases (CRLM). Currently, HGPs are determined postoperatively. In this study, we evaluated radiomics for preoperative prediction of HGPs on computed tomography (CT), and its robustness to segmentation and acquisition variations. Patients with pure HGPs [i.e. 100% desmoplastic (dHGP) or 100% replacement (rHGP)] and a CT-scan who were surgically treated at the Erasmus MC between 2003–2015 were included retrospectively. Each lesion was segmented by three clinicians and a convolutional neural network (CNN). A prediction model was created using 564 radiomics features and a combination of machine learning approaches by training on the clinician’s and testing on the unseen CNN segmentations. The intra-class correlation coefficient (ICC) was used to select features robust to segmentation variations; ComBat was used to harmonize for acquisition variations. Evaluation was performed through a 100 × random-split cross-validation. The study included 93 CRLM in 76 patients (48% dHGP; 52% rHGP). Despite substantial differences between the segmentations of the three clinicians and the CNN, the radiomics model had a mean area under the curve of 0.69. ICC-based feature selection or ComBat yielded no improvement. Concluding, the combination of a CNN for segmentation and radiomics for classification has potential for automatically distinguishing dHGPs from rHGP, and is robust to segmentation and acquisition variations. Pending further optimization, including extension to mixed HGPs, our model may serve as a preoperative addition to postoperative HGP assessment, enabling further exploitation of HGPs as a biomarker.

**Supplementary Information:**

The online version contains supplementary material available at 10.1007/s10585-021-10119-6.

## Introduction

Colorectal liver metastases (CRLM) represent approximately 30% of all metastases in patients with colorectal carcinoma [1]. Ten-year survival after CRLM resection is 20%, primarily limited due to recurrent disease [2]. Prognosis estimation is challenging since powerful prognosticators are lacking.

Histopathological growth patterns (HGPs) have recently been identified as independent prognosticators in patients after CRLM resection [3]. The interface between tumor cells and normal liver parenchyma (NLP) is characterized by three distinct HGPs: two frequent (desmoplastic HGP (dHGP) and replacement HGP (rHGP), see Supplementary Fig. S1) and one rare (pushing HGP) type [4, 5]. A previous study found that dHGP patients have superior survival compared to mixed, replacement or pushing HGP patients [3]. Moreover, recent studies have suggested that HGPs could predict systemic chemotherapy effectiveness [6, 7]. Previous guidelines suggested a cut-off of 50% of a single HGP to determine the dominant HGP [4]. More recent studies have shown that pure HGPs (i.e., 100% of the interface expresses the HGP) appear clinically more relevant [8].

Preoperative HGP assessment is currently not possible, as assessment requires pathology slices of resection specimens to be reviewed with a light microscope. Biopsy material is not suitable due to lesion heterogeneity. Preoperative assessment, however, could provide valuable information on prognosis, could help identifying patients who benefit from perioperative systemic treatment, and could be used to evaluate response treatment by monitoring changes in the HGP [6, 7]. As there is currently no method to assess HGPs preoperatively, investigating these potential improvements is not possible. Hence there is a need to identify HGPs based on medical imaging to exploit the full potential of HGPs as a biomarker, as concluded by a recent review [9].

The field of radiomics has emerged as a non-invasive way to establish relations between quantitative image features and tumor biology or clinical outcomes [10]. Several radiomics studies have shown promising results in a wide variety of applications [11]. In CRLM, radiomics has been used to assess chemotherapy response, survival, detect CRLM, and predict mixed HGPs [12–15]. A major drawback of many radiomics approaches is the dependence on manual segmentations, which may introduce observer variability in the predictions [16–18]. Additionally, image acquisition variations may affect the predictions [19].

The primary aim of this study was to evaluate if radiomics can preoperatively distinguish pure HGPs on computed tomography (CT) scans as a non-invasive addition to postoperative histological assessment, enabling pre-operative treatment response prediction and evaluation. The secondary aim was to evaluate and improve the robustness of the radiomics models to variations in segmentation and acquisition protocol.

## Methods and materials

### Patients

This study was performed in accordance with the Dutch Code of Conduct for Medical Research of 2004 and approved by the local institutional review board (“Medische Ethische Toetsings Commissie” (METC), MEC-2017-479). As the study was retrospectively performed with anonymized data, the need for informed consent was waived. Patients surgically treated at the Erasmus MC between 2003–2015 with a preoperative CT-scan in the portal venous phase (PVP) and available hematoxylin and eosin stained tissue sections were included retrospectively. Patients with recurrent CRLM or CRLM requiring two-staged resections were not included. Both synchronous and metachronous resections were allowed. Pre-contrast and arterial phase CT were available in a minority of patients and therefore excluded. Patients treated with preoperative chemotherapy were excluded, since chemotherapy may alter HGPs [3]. HGPs were scored on resection specimens according to the consensus guidelines by an expert pathologist (PV) [5]. In this pilot, we focused on pure HGPs as these appear clinically more relevant than mixed HGPs, as a previous study showed that pure dHGP is an unmatched predictor for improved survival in chemo-naïve patients with CRLM [8]. Furthermore, we hypothesized that the use of radiomics has a higher chance of success in distinguishing pure HGPs, as their morphology is less heterogeneous than mixed HGPs. Patients with pure pushing HGPs were excluded, as this is rare (< 1%) [4–6, 8]. The pure dHGPs and rHGPs both make up about 20% of the total population of chemo-naive patients, resulting in inclusion of 40% of all available patients [8].

Various clinical characteristics were collected: age, sex, primary tumor location and nodal status, disease free interval between resection of the colorectal carcinoma and CRLM detection, and the preoperative carcinoembryonic antigen level. Size and number of CRLMs, including ablations without histology, were derived from the CT-scans.

### Segmentation

Lesion segmentation was independently performed by four observers: a medicine student with no relevant experience (STUD1), a PhD student (PhD) with limited experience, an expert abdominal radiologist (RAD), and an automatic CNN. The student segmented all lesions within a week, and immediately afterwards, segmented all lesions a second time (STUD2) to evaluate the intra-observer variability. As the order of segmentation was not the same in the first and second time, but randomized, the time between the first and second segmentation varied between two and seven days. Segmentation agreement between all observer pairs was determined through the pairwise dice similarity coefficient (DSC).

Segmentation by the clinicians was performed with in-house Python-based software [20]. For the lesions, the clinicians could segment manually or semi-automatically using region-growing or slice-to-slice contour propagation. Segmentation was performed per slice in the 2D transverse plane, resulting in a 3D volume. Semi-automatic results were always reviewed by the individual clinicians and manually corrected when necessary to assure the result resembled manual segmentation.

The Hybrid-Dense-UNet, which achieved state-of-the-art performance on the liver tumor segmentation (LITS) challenge and is open-source, was used to automatically segment the NLP and lesions [21, 22]. The original CNN as trained on the LITS data that was published open-source was used. Lesions which were segmented by the CNN but had no histology were excluded. For lesions that were not segmented by the CNN, but for which histology was available, the segmentation of the radiologist (RAD) was used, resembling implementation in clinical practice. As the Hybrid-Dense-UNet was trained to simultaneously segment the NLP and lesions, this CNN was also used to segment the NLP [21].

### Radiomics

**Fig. 1:**
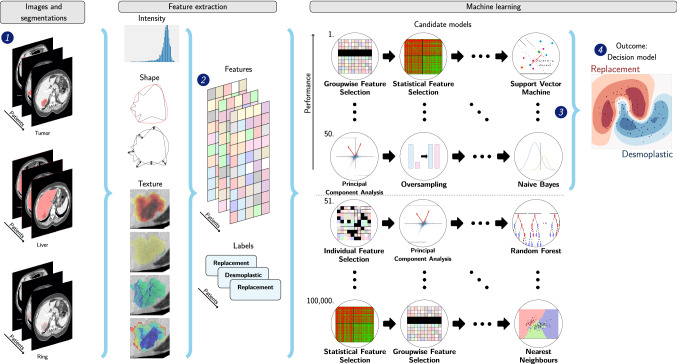
Schematic overview of the radiomics approach: adapted from [24]. Processing steps include segmentation of the lesion and liver, and extraction of the lesion ring (1), feature extraction from the CT based on these regions (2), and the creation of a decision model from the features (4), using an ensemble of the best 50 workflows from 100,000 candidate workflows (3), where the workflows are different combinations of the different processing and analysis steps (e.g. the classifier used). (Color figure online)

From each region of interest (ROI) on the CT, 564 radiomics features were extracted. Features were extracted per segmentation, e.g. for each 3D ROI by each observer. Details can be found in Supplementary Materials A. Based on these features, decision models were created using the workflow for optimal radiomics classification (WORC) toolbox, see Fig. 1 [23–25]. In WORC, decision model creation consists of several steps, e.g. feature scaling, selecting relevant features, and classification with machine learning. WORC performs an automated search among a variety of algorithms for each step and determines which combination of algorithms maximizes the prediction performance on the training set. For example, in the machine learning step, one of the eight following algorithms may be used: (1) logistic regression; (2) support vector machines; (3) random forests; (4) naive Bayes; (5) linear discriminant analysis; (6) quadratic discriminant analysis; (7) AdaBoost [26]; and (8) extreme gradient boosting [27]. Details can be found in Supplementary Materials B. The code including all parameters for our experiments has been published open-source [28].

### Robustness to segmentation and image acquisition variations

Robustness to segmentation variations was assessed using the intra-class correlation coefficient (ICC) of the features, defining good (ICC > 0.75) and excellent (ICC > 0.90) reliability [29]. Moreover, the impact of ICC-based feature selection on model performance was assessed by creating models using only these features.

Robustness to variations in the acquisition parameters was assessed by using ComBat [30, 31]. In ComBat, feature distributions are harmonized for variations in the imaging acquisition, e.g. due to differences in hospitals, manufacturers, or acquisition parameters. When dividing the dataset into groups based on these variations, the groups have to remain sufficiently large to estimate the harmonization parameters. In our study, groups were defined based on manufacturer alone or combined with slice thickness (above or below the median) without a moderation variable.

### Experimental setup

For each experiment, a 100 × random-split cross-validation [32, 33] was performed, randomly splitting the data in each iteration in 80% for training and 20% for testing, see Supplementary Fig. S2. In each iteration, a second, internal 5 × random-split cross-validation was performed on the training set, using 85% for training and 15% for validation, where the validation sets were used to optimize the model hyperparameters. Hence, in each iteration, we enforced a strict separation into training, validation and test sets: model construction was performed automatically within the training and validation sets, leaving the test set untouched to minimize the chance of overfitting. The splitting was stratified to maintain a similar dHGP/rHGP ratio in all datasets. Lesions of a patient belonged either all to the training or all to the test dataset.

First, four single-observer radiomics models were created, each using the segmentations of a different observer (STUD2, PhD, RAD, and CNN), but keeping the same observer for training and testing.

**Fig. 2:**
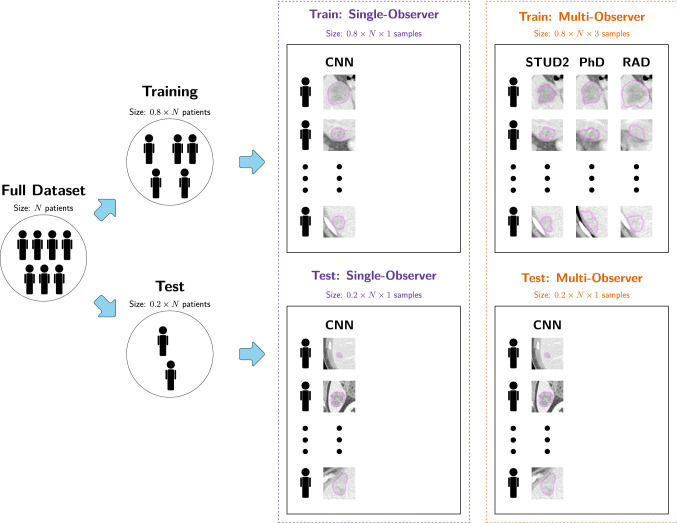
Schematic overview of the evaluation setup in a single random-split cross-validation iteration for the single-observer and multi-observer models. For the single-observer models, here illustrated for observer CNN, for both the patients included in the training and in the testing set, each patient appears one time with the segmentation of that single observer. For the multi-observer model, the test set is exactly the same as the single-observer model. However, in the training set, each patient appears three times, each time with a different segmentation from one of the three other observers (STUD2, PhD, and RAD). Hence, in the multi-observer model, the training set size is effectively tripled compared to the single-observer model, while the test set remains unchanged. (Color figure online)

Second, a multi-observer radiomics model was trained with segmentations of three observers (STUD2, PhD, and RAD) and tested with segmentations of the fourth, unseen observer (CNN). We hypothesized that a model trained on segmentations from multiple observers may yield a higher performance, and a higher robustness to segmentation variations, as the model is forced to find characteristics shared by all segmentations. For the multi-observer model, the data was split per patient into training and test sets in the same way as in the single-observer model, see Fig. 2. However, each lesion included in the training set appeared three times, each time with a different segmentation from one of the three observers. The number of training samples was therefore increased to a threefold of the number of training samples used for the single-observer model. This can be seen as a form of data augmentation [34], as compared to the single-observer model, the number of training samples is increased by adding slightly modified copies of the original training samples. Each lesion included in the test set appeared only once, using the segmentation of the CNN.

Third, to estimate model robustness to segmentation and acquisition protocol variations, additional multi-observer models were created using only reliable features (good or excellent) through ICC-based feature selection and ComBat, respectively.

Lastly, features extracted from three other ROIs were evaluated: NLP, and based on the multi-observer setup, NLP plus the lesion, and the lesion border [3, 8], see Supplementary Fig. S3. Also, to evaluate the predictive value of the clinical characteristics (i.e., 1: age; 2: sex; 3: primary tumor location; 4: primary tumor nodal status; 5: disease free interval; 6: preoperative carcinoembryonic antigen level; 7: CRLM size; and 8: number of CRLMs), two additional HGP prediction models were evaluated using: (1) clinical characteristics (“single-observer”); and (2) imaging and clinical characteristics.

### Statistics

The individual predictive values of the radiomics features and the clinical characteristics, and the differences in CT acquisition parameters, were assessed using a Mann–Whitney U test for continuous variables, and a Chi-square test for categorical variables. To this end, the radiomics features extracted from the CNN segmentations were used, as these segmentations were used in the test set in the multi-observer models. The p-values of the radiomics features were corrected for multiple testing using the Bonferroni correction (i.e., multiplying the p-values by the number of tests). All p-values were considered statistically significant at a p-value ≤ 0.05.

Performance was evaluated in the test dataset using accuracy, area under the curve (AUC) of the receiver operating characteristic (ROC) curve, sensitivity, and specificity, averaged over the 100 × cross-validation iterations. The corrected resampled t-test was used to construct 95% confidence intervals (CIs), taking into account that the samples in the cross-validation splits are not statistically independent [33]. ROC confidence bands were constructed using fixed-width bands [35]. The positive class was defined as dHGP. The performance estimates in the training dataset are not reported, as these would be too optimistic, since the used methods tend to over-fit on the training dataset [36].

## Results

### Dataset

The dataset included 93 lesions (46 dHGP; 47 rHGP) of 76 patients (Table 1). The median age was 68 years (interquartile range 60–76 years). No statistically significant differences in clinical characteristics between dHGP and rHGP CRLM were found.

**Table 1:** Patient and imaging characteristics of the 76 patients included in this study. P-values are calculated using a Mann–Whitney U test for continuous variables, a chi-square test for continuous variables

Patients	All	Desmoplastic	Replacement	p-value
Total	76	37 (48.0%)	39 (52.0%)	0.82
Age^†^	68.0 (59.5–75.5)	68.0 (60.0–75.5)	68.0 (59.0–77.0)	
Sex				0.23
Male	44 (57.9%)	24 (64.9%)	20 (51.3%)	
Female	32 (42.1%)	13 (35.1%)	19 (48.7%)	
Primary tumor location				0.56
Right-sided	6 (8.3%)	2 (5.7%)	4 (10.8%)	
Left-sided	29 (54.2%)	21 (60.0%)	18 (48.6%)	
Rectum	27 (37.5%)	12 (34.3%)	15 (40.5%)	
Missing	4			
Nodal status primary tumor				0.66
N0	35 (46.1%)	18 (48.6%)	17 (43.6%)	
N +	41 (53.9%)	19 (51.4%)	22 (56.4%)	
Disease free interval				0.64
≤ 12 months	37 (48.7%)	17 (45.9%)	20 (51.3%)	
> 12 months	39 (51.3%)	20 (51.4%)	19 (48.7%)	
Number CRLM				0.51
≤ 1	54 (71.1%)	25 (67.6%)	29 (74.4%)	
> 1	22 (28.9%)	12 (34.4%)	10 (25.6%)	
Size largest CRLM				0.63
≤ 5 cm	60 (81.1%)	30 (83.3%)	30 (78.9%)	
> 5 cm	14 (18.9%)	6 (16.7%)	8 (21.1%)	
Missing	2			
CEA*				0.21
≤ 200 µg/L	65 (92.9%)	32 (97.0%)	33 (89.2%)	
> 200 µg/L	5 (7.1%)	1 (3.0%)	4 (10.8%)	
Missing	6			
Imaging				
Slice thickness (mm)^†^	5.0 (3.0–5.0)	4.0 (3.0–5.0)	5.0 (3.0–5.0)	0.40
Pixel spacing (mm)^†^	0.74 (0.68–0.78)	0.78 (0.71–0.78)	0.71 (0.67–0.75)	**0.007**
Tube current (mA)^†^	239 (143–325)	239 (151–305)	232 (135–332)	0.38
Peak kilovoltage^†^	120 (120–120)	120 (120–120)	120 (120–120)	0.09

P-values in bold are deemed significant (< 0.05)

*Abbreviations: *CEA* carcinoembryonic antigen; *CRLM* colorectal liver metastases; *IQR* interquartile range

^†^Values are median (interquartile range). Other values than those given in the median and interquartile range may occur

Since the Erasmus MC serves as a tertiary referral, the CT-scans originated from 37 different scanners, resulting in considerable acquisition protocol variations (Table 1). The differences in acquisition parameters were not statistically significant, except for pixel spacing (p = 0.007, median of 0.78 vs. 0.71 mm). Additionally, nineteen different reconstruction kernels were used, and four manufacturers were present (Siemens: 43, Philips: 16, Toshiba: 16, General Electric: 1).

### Segmentation

**Fig. 3:**
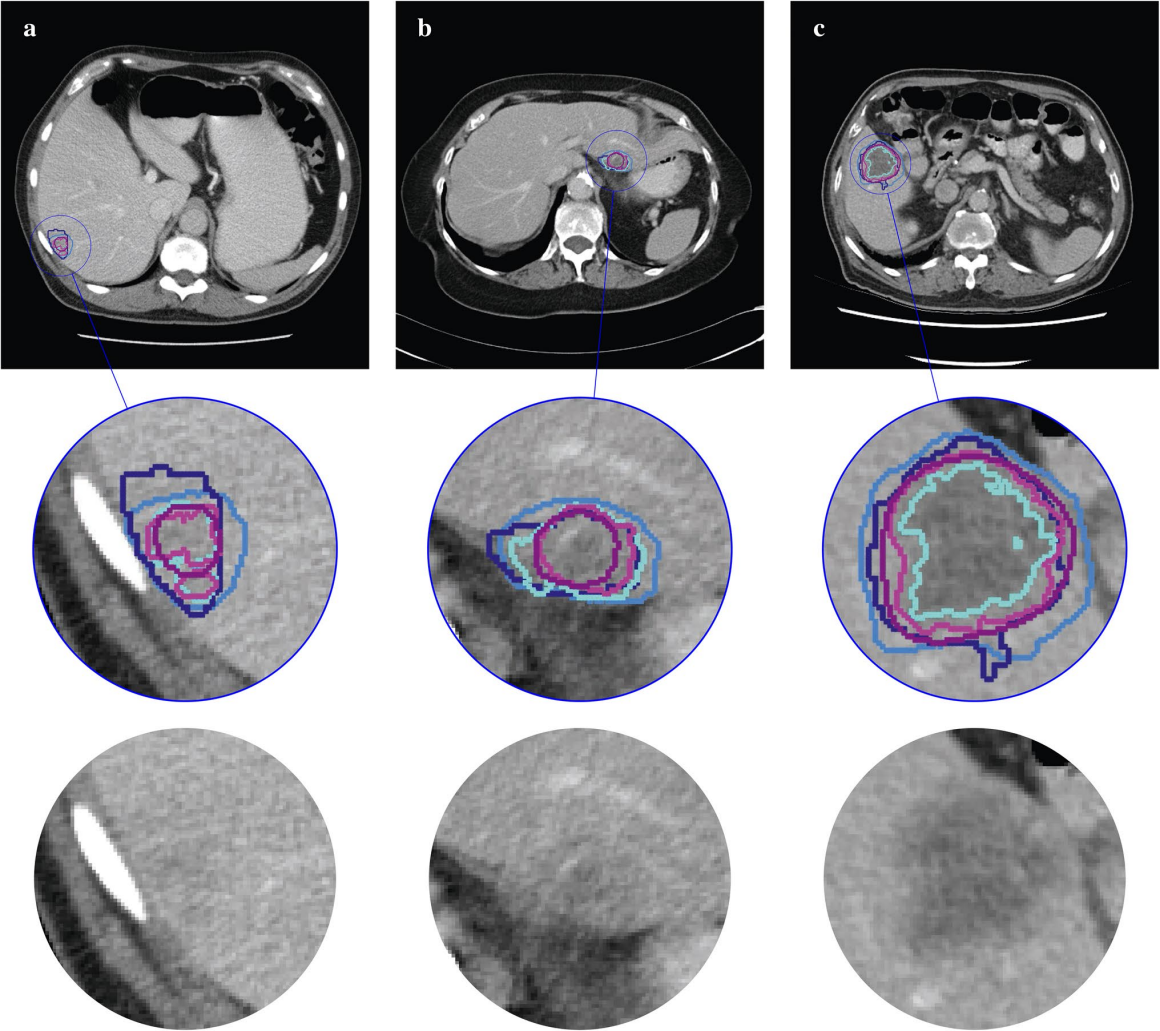
Examples of segmentations of three colorectal liver metastases (CRLMs) by the human observers and by the convolutional neural network (CNN) [PhD (dark blue); RAD (light blue); STUD first try (STUD1) (cyan) and second try (STUD2) (magenta); CNN (purple)] on a single axial slice of CT-scans. The bottom row depicts the zoomed in region without the segmentation overlays. The three CRLMs displayed are those with a volume at the 25% percentile (**a**), 50% percentile (**b**) and 75% percentile (**c**) of all metastases in the database. (Color figure online)

Lesion segmentation examples are presented in Fig. 3. The CNN failed to detect 8 of the 93 included lesions (9%), for which the radiologist’s segmentation was used. The pairwise DSC to assess the observer segmentation agreement is shown in Supplementary Table S1. The intra-observer agreement (DSC of 0.80 for STUD1 and STUD2) was higher than the inter-observer agreement (mean DSC of 0.69 for all other human observers).

### Radiomics

In Table 2, the performance of the four single-observer models is shown. The mean AUC of all models was above random guessing (0.50), but varied per observer [STUD2: 0.69 (95% CI 0.56–0.82), PhD: 0.66 (95% CI 0.53–0.79), RAD: 0.72 (95% CI 0.59–0.83), and CNN: 0.66 (95% CI 0.54–0.79)]. As the 95% confidence intervals showed substantial overlap, the differences were not statistically significant. Hence, in terms of AUC, the models performed similarly.

**Table 2:** Performance of the radiomics models using segmentations from single observers (STUD2, PhD, RAD, and CNN) both for the patients in the training sets and the other patients in the test sets

	STUD2	PhD	RAD	CNN
AUC	0.69 [0.56, 0.82]	0.66 [0.53, 0.79]	0.72 [0.59, 0.83]	0.66 [0.54, 0.79]
Accuracy	0.65 [0.55, 0.75]	0.61 [0.50, 0.71]	0.65 [0.55, 0.76]	0.62 [0.52, 0.72]
Sensitivity	0.64 [0.49, 0.80]	0.57 [0.41, 0.72]	0.62 [0.49, 0.76]	0.61 [0.45, 0.76]
Specificity	0.65 [0.48, 0.82]	0.65 [0.49, 0.81]	0.68 [0.52, 0.85]	0.63 [0.47, 0.78]

For each metric, the mean and 95% confidence interval over the 100 × random-split cross-validation iterations on the test sets are given

*Abbreviations: *AUC* area under the receiver operator characteristic curve

**Fig. 4:**
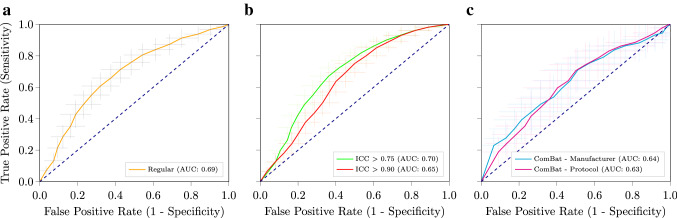
Receiver operating characteristic (ROC) curves of the radiomics models using segmentations from multiple observers (STUD2, PhD, and RAD) for the patients in the training sets and the segmentations from another observer (CNN) in the other patients in the test sets. These include the regular model (**a**); using only features with an intra-class correlation coefficient (ICC) larger than 0.75 or 0.90 (**b**); and using ComBat to harmonize differences in manufacturer or protocol (**c**). The crosses indicate the 95% confidence intervals; the curves the means. The dashed lines indicate the performance of random guessing. (Color figure online)

In Table 3 and Fig. 4, the multi-observer model performance is shown. Performance was similar [mean AUC of 0.69 (95% CI 0.57–0.81)] to the single-observer models (Fig. 4a). Using only features with good (N = 263) [mean AUC of 0.70 (95% CI 0.59–0.81)] or excellent reliability (N = 166) [mean AUC of 0.65 (95% CI 0.53–0.77)] across the human observers did not improve the performance (Fig. 4b). Using ComBat to harmonize the features for manufacturer [mean AUC of 0.64 (95% CI 0.40–0.88)] or protocol [mean AUC of 0.63 (95% CI 0.38–0.87)] differences yielded a minor performance decrease (Fig. 4c). As there was only one General Electric scan, this scan was omitted from harmonization.

**Table 3:** Performance of the radiomics models using segmentations from multiple observers (STUD2, PhD, and RAD) for the patients in the training sets and the segmentations from another observer (CNN) in the other patients in the test sets

	Regular	ICC > 0.75	ICC > 0.90	ComBat Man	ComBat Prot
AUC	0.69 [0.57, 0.81]	0.70 [0.59, 0.81]	0.65 [0.53, 0.77]	0.64 [0.40, 0.88]	0.63 [0.38, 0.87]
Accuracy	0.65 [0.54, 0.76]	0.65 [0.55, 0.75]	0.61 [0.50, 0.72]	0.60 [0.41, 0.79]	0.58 [0.39, 0.76]
Sensitivity	0.71 [0.57, 0.86]	0.63 [0.48, 0.78]	0.61 [0.44, 0.77]	0.56 [0.30, 0.82]	0.55 [0.29, 0.81]
Specificity	0.58 [0.41, 0.74]	0.67 [0.51, 0.83]	0.61 [0.45, 0.78]	0.63 [0.33, 0.93]	0.60 [0.29, 0.90]

The performance is reported for: the regular model; using only features with good (ICC > 0.75) or excellent (ICC > 0.90) reliability; and using ComBat harmonization per manufacturer (Man) or per acquisition protocol (Prot) without a moderation variable. For each metric, the mean and 95% confidence interval over the 100 × random-split cross-validation iterations are given

*Abbreviations: *AUC* area under the receiver operator characteristic curve; *ICC* intra-class correlation coefficient; *Man* manufacturer; *Prot* protocol

Table 4 contains the performances of the models trained on other features, including NLP [mean AUC of 0.65 (95% CI 0.51–0.78)], and based on the multi-observer setup, NLP plus the lesion [mean AUC of 0.63 (95% CI 0.52–0.75)] and the lesion border [mean AUC of 0.67 (95% CI 0.56–0.78)]. Hence, the performance was (slightly) worse than using only lesion features. The model based on clinical characteristics performed similarly to random guessing [mean AUC of 0.56 (95% CI 0.43–0.70)]: the model trained on clinical characteristics plus lesion features performed worse than lesion-only [mean AUC of 0.65 (95% CI 0.53–0.77)].

**Table 4:** Performance of models using features other than only lesion features

Metric	NLP	NLP + Lesion	Ring	Clinical	Clinical + Lesion
AUC	0.65 [0.51, 0.78]	0.63 [0.52, 0.75]	0.67 [0.56, 0.78]	0.56 [0.43, 0.70]	0.65 [0.53, 0.77]
Accuracy	0.59 [0.49, 0.70]	0.60 [0.50, 0.71]	0.63 [0.54, 0.73]	0.53 [0.41, 0.64]	0.62 [0.51, 0.72]
Sensitivity	0.52 [0.33, 0.70]	0.60 [0.43, 0.76]	0.67 [0.51, 0.83]	0.56 [0.37, 0.75]	0.62 [0.45, 0.79]
Specificity	0.67 [0.50, 0.85]	0.61 [0.46, 0.75]	0.59 [0.45, 0.74]	0.49 [0.31, 0.67]	0.61 [0.45, 0.77]

These features were extracted from a segmentation of the normal liver parenchyma (NLP); the NLP and the lesion (NLP + Lesion); a ring at the border of the segmentation (Ring); using the clinical characteristics (Clinical); and the clinical characteristics combined with lesion features (Clinical + Lesion)

*Abbreviations: *AUC* area under the receiver operating characteristic curve; *NLP* normal liver parenchyma; *CNN* convolutional neural network

After Bonferroni correction for multiple testing, from the 564 features extracted using the CNN segmentations, only four texture features derived from Gabor filters were found to have statistically significant p-values (0.035–0.010).

## Discussion

The aim of this pilot was to evaluate whether radiomics can distinguish pure dHGPs from pure rHGPs based on CT-scans and to evaluate its robustness to segmentation and acquisition protocol variations. Despite these variations, our results suggest that radiomics features have predictive value in distinguishing pure HGPs on CT-scans, but that caution is warranted when drawing conclusions about the clinical applicability at this stage.

Currently, HGPs can only be determined after surgery using resection specimens. Our radiomics approach may overcome this gap. Preoperative HGP assessment may give an earlier estimate of disease aggressiveness and prognosis, thus improving patient care [9]. A previous study found a 5-year overall survival of 78% in dHGP patients compared to 37% (p < 0.001) in patients with other HGPs [8]. Preoperative assessment of HGPs may even imply a practice change, as HGPs may be associated with efficacy of systemic chemotherapy [3, 6–8]. Hence, preoperative HGP assessment through radiomics may also be used predictively to select patients which may benefit from chemotherapy. Moreover, preoperative HGP assessment may enable others to study the full potential of HGP as a biomarker [9]. Although it is difficult at this stage to decide on the accuracy of radiomics-based HGP prediction required for clinical practice, the current performance is likely not sufficient yet and further improvements are warranted.

Our secondary aim was to evaluate and improve the robustness of radiomics to segmentation and acquisition protocol variations. Our results indicate substantial differences between the segmentations. In spite of these differences, our multi-observer model generalized well to segmentations of an unseen “observer”, i.e., the automated CNN. Generally, improving model robustness to segmentation variations is done by selecting only reliable features, i.e., high ICC across multi-observer segmentations [16–18]. However, in our results, this did not alter the performance, indicating that training on multiple observers already enforced model robustness to segmentation variations. As the unseen observer was a CNN, our combined approach (CNN for segmentation, radiomics for classification) is fully automatic and observer independent. It must be pointed out that, although we used a state-of-the-art CNN ranking second in the renowned LITS challenge [22], 8 lesions (9%) were missed by the CNN. These required manual correction, making the method actually semi-automatic in this minority of cases. The radiologist however initially also missed 19 lesions (20%), which were later corrected based on the pathology outcome, indicating that human observers also miss lesions. Of these 19 lesions, 16 were detected by the CNN. This indicates that the CNN may aid identifying false negatives from the radiologists. However, the CNN detected 257 abnormalities in total, likely including a large number of false positives, which would require correction by the radiologist. Future studies should systematically compare the hit and miss ratios of radiologists and the CNN. Nonetheless, we believe the method’s large degree of automation and its observer independence are highly desirable aspects for use in clinical practice.

Visual inspection of the lesions indicated that the radiologist’s segmentations showed the largest difference with the CNN segmentations. In addition, the radiologist’s segmentations had the lowest overlap (in terms of DSC) with the other observers. Visual inspection indicated that the radiologist generally drew a loose outline around the lesion, and thus ROIs with a relatively large area, while the CNN generally drew conservative outlines, thus ROIs with a relatively small area. Caution should be taken when drawing conclusions, as we only compared ROIs of a single radiologist with the CNN. Moreover, as annotating lesion boundaries is not part of routine clinical practice of radiologists, their segmentations cannot be considered as the ground truth.

Additionally, we evaluated models using features extracted from several ROIs to investigate where the most relevant HGP information is. The NLP model performed worse than the lesion-only models. As HGPs are represented at the liver tissue and lesion interface, we expected the combination or usage of the border to be optimal. However, combining these features, or using the border, did not yield an improvement over the lesion-only model. This may be attributed to the fact that determination of the exact border of the lesion is difficult. Our radiomics model uses a more data-driven approach, using 564 features extracted not only from the lesion boundary but from the full lesion segmentation, and machine learning to determine what information is most relevant. Our results suggest that the lesion itself contains the most informative features. The clinical characteristics did not yield any predictive value on their own, nor added predictive value when combined with the radiomics features. This is in line with the literature, as to our knowledge, no pre-operative biomarkers for HGPs based on clinical characteristics have so far been described [9].

Recently, the value of radiomics to predict HGPs was assessed by Cheng et al. (2019) [16] using the former consensus guidelines [4]. This study included 126 CRLMs, using for each patient a pre- and post-contrast arterial and PVP CT-scan. An AUC of 0.93 in the training and 0.94 in the validation set was reported, which was much higher than the performance in our study. This difference may be attributed to various factors in the study design. First, we used the more recent clinical guidelines and included only pure HGPs, instead of the previous cut-off of > 50% of a single HGP [4, 8]. There may be considerable uncertainty in the scoring of pure HGPs, e.g. other HGP types may be missed due to sampling errors [4]. Some cases could be misclassified due to this possible missing information, limiting our performance. Second, Cheng et al. (2019) [16] used multiple CT-scans per patient: an AUC of 0.79 was obtained in the used validation set when only using the PVP, as we did. Also, we used a multi-center CT dataset with much acquisition protocol heterogeneity, while Cheng et al. (2019) [16] used a two-center dataset with comparable acquisition protocols. Moreover, our radiomics approach is different, e.g. we used a fully automatic approach optimized on the training set, while the optimization protocol used by Cheng et al. (2019) is not explicitly mentioned.

There are several limitations to this study. First, our dataset included only pure dHGP or rHGP patients, while mixed and a rare third HGP (pushing) exist as well. The strict selection resulted in a small sample size, which may explain the wide CIs. Due to the large width of the CIs, i.e., the AUCs generally spanned between 15–30% of the range, few claims could be made regarding statistical significance of differences between models. No claims can be made about the performance of the model on mixed HGPs or the pushing HGP. Future studies should include mixed HGPs, which will lead to a larger dataset, and will improve clinical applicability.

Second, we used PVP contrast-enhanced CT-scans, as this was mostly used in clinical routine. Addition of other contrast phases, positron emission tomography or magnetic resonance imaging, may improve the performance [16, 37, 38].

Third, while our CNN produced segmentations similar to the human observers as indicated by the DSC, 8 out of the 93 included lesions were missed. As the CNN segmentations are similar to those of the radiologist and our multi-observer model is robust to segmentation variations, replacing the missed segmentations with the radiologist’s is not expected to have substantially influenced our results.

Lastly, our imaging models were trained and evaluated on a multi-center, heterogeneous dataset. On one hand, this is a strength of our study, as the models had predictive value despite substantial acquisition variations. However, heterogeneity may have (negatively) affected our performance. The use of ComBat to compensate for manufacturer variations did not lead to a substantial improvement in prediction accuracy. Additional experiments with ComBat using the HGP as a “moderation variable” showed a near perfect performance; however, such use of the HGP as a moderation variable in the ComBat algorithm is a form of overfitting, as it uses the ground truth HGP data of the full dataset (including the test set), and it tends to give too optimistic performance estimates. Future research could explore other methods to compensate for manufacturer variations on the one hand while maintaining the distinction between HGPs on the other hand. Alternatively, using a single-scanner study will limit the generalizability, but may positively impact the performance. Additionally, although we used a multi-center dataset, we did not perform an independent, external validation. However, we used a rigorous cross-validation, separating the data 100 × in training and testing parts. Hence, as our radiomics approach was optimized on the training set only, the chance of overestimating performance due to “over-engineering” was limited.

Future research could include HGP classification using CNNs. While our current method is largely observer independent, classification without use of any segmentation would be truly observer independent. Also, only four lesion feature showed statistically significant differences between the dHGP and rHGP lesions, suggesting that these features may not be optimal for distinguishing these HGPs. The CNN used for segmentation in our study was not designed for HGP prediction, but rather segmentation of the liver and various liver abnormalities. Features learned by a dedicated classification CNN for HGP prediction may yield more predictive value than the features learned by our segmentation CNN or the generic radiomics features used in our study. This would probably require a larger dataset to learn from.

## Conclusions

Our combination of deep learning for segmentation and radiomics for classification shows potential for automatically distinguishing pure dHGPs from rHGPs of CRLM on CT-scans. The model is observer independent and robust to segmentation variations. However, the current performance is likely not sufficient yet and further improvements are warranted, including extension to mixed HGPs, and external validation. Pending further optimization, radiomics may serve as a non-invasive, preoperative addition to postoperative HGP assessment, enabling pre-operative response prediction, response evaluation, and further studies on HGP as a pre-operative biomarker.

## Supplementary Information

Below is the link to the electronic supplementary material.

Supplementary file1: Supplementary Materials (DOCX 45 kb)

Supplementary file1 (Table S1): Segmentation agreement expressed in Dice Similarity Coefficient (DSC) (mean (standard deviation)) between the observers and the convolutional neural network (CNN) (STUD (1st and 2nd time), PhD, RAD, CNN). The average of the mean and standard deviation of the DSC for each observer are stated in the bottom row (DOCX 13 kb)

Supplementary file6 (Table S2): Overview of the 564 features used in this study. GLCM features were calculated in four different directions (0, 45, 90, 135 degrees) using 16 gray levels and pixel distances of 1 and 3. LBP features were calculated using the following three parameter combinations: 1 pixel radius and 8 neighbours, 2 pixel radius and 12 neighbours, and 3 pixel radius and 16 neighbours. Gabor features were calculated using three different frequencies (0.05, 0.2, 0.5) and four different angles (0, 45, 90, 135 degrees). LoG features were calculated using three different widths of the Gaussian (1, 5 and 10 pixels). Vessel features were calculated using the full mask, the edge, and the inner region. Local phase features were calculated on the monogenic phase, phase congruency and phase symmetry (DOCX 17 kb)

Supplementary file2 (Figure S1): Replacement type (A) and desmoplastic type (B) histopathological growth pattern on hematoxylin and eosin stained tissue sections (PDF 4812 kb)

Supplementary file3 (Figure S2): Visualization of the 100x random split cross-validation, including a second cross-validation within the training set for model optimization. The test dataset is only used for evaluation of the trained model (PDF 61 kb)

Supplementary file4 (Figure S3): Examples of segmentations of various regions of interest on a single axial slice of CT-scans. A: CT-scan without segmentation; B: lesion; C: normal liver parenchyma; and D: ring on the border between the lesion and normal liver parenchyma (PDF 655 kb)

## Data Availability

Imaging and clinical research data are not available at this time. Programming code is available on Zenodo at https://doi.org/10.5281/zenodo.4392829.

## Abbreviations

AUC: Area under the receiver operating characteristic curve
CI: Confidence interval
CNN: Convolutional neural network
CRLM: Colorectal liver metastases
CT: Computed tomography
DSC: Dice similarity coefficient
HGP: Histopathological growth pattern
rHGP: Replacement histopathological growth pattern
dHGP: Desmoplastic histopathological growth pattern
ICC: Intra-class correlation coefficient
NLP: Normal liver parenchyma
PVP: Portal venous phase
ROC: Receiver operating characteristic
ROI: Region of interest
