# Supplementary material for: Distinguishing pure histopathological growth patterns of colorectal liver metastases on CT using deep learning and radiomics: a pilot study
Source: Clin Exp Metastasis. 2021 Sep 17;38(5):483–94. doi: 10.1007/s10585-021-10119-6 (PMC8510954; doi:10.1007/s10585-021-10119-6)
Supplement: Supplementary file 1 — Supplementary file1: Supplementary Materials (DOCX 45 kb) [file 10585_2021_10119_MOESM1_ESM.docx]

**Distinguishing pure histopathological growth patterns of colorectal liver metastases on CT using deep learning and radiomics: a pilot study**

Martijn P. A. Starmans *MS* *^,^ *^†,^* ^1^, Florian E. Buisman *MD* *^†,^* ^2^, Michel Renckens *MD* ^2^, François E. J. A. Willemssen *MD* ^1^, Sebastian R. van der Voort *MS* ^1^, Bas Groot Koerkamp *MD PhD* ^2^*,* Dirk J. Grünhagen *MD PhD* ^2^, Wiro J. Niessen *PhD* ^1,3^, Peter B. Vermeulen *PhD ^4^,* Cornelis Verhoef *MD* *PhD* ^2^, Jacob J. Visser *MD* *PhD* ^1^ and Stefan Klein *PhD* ^1^

*^1^Department of Radiology and Nuclear Medicine, Erasmus MC, Rotterdam, the Netherlands*

*^2^Department of Surgery, Erasmus MC Cancer Institute, Rotterdam, the Netherlands*

*^3^Faculty of Applied Sciences, Delft University of Technology, Delft, the Netherlands*

*^4^Translational Cancer Research Unit, Department of Oncological Research, Oncology Center, GZA Hospitals Campus Sint-Augustinus and University of Antwerp, Antwerp, Belgium*

**Corresponding author: Martijn P. A. Starmans* ([m.starmans@erasmusmc.nl](mailto:m.starmans@erasmusmc.nl))

*^†^These authors contributed equally*

**Supplementary Materials**

**Supplementary Materials A. Feature Extraction**

This supplementary material is similar to Vos et al. (2019) [1] and Timbergen et al. (2020) [2], but details relevant for the current study are highlighted.

A total of 564 radiomics features quantifying intensity, shape, orientation and texture were extracted. These features were extracted using the defaults for CT scans from the Workflow for Optimal Radiomics Classification (WORC) toolbox [3], which internally uses the PREDICT [4] and PyRadiomics [5] feature extraction toolboxes. The code to extract the features for this specific study has been published open-source [6]. An overview of all features is depicted in **Supplementary Table S2**. For details on the mathematical formulation of the features, we refer the reader to Zwanenburg et al. (2020) [7]. More details on the extracted features can be found in the documentation of the respective toolboxes, mainly the WORC documentation [8].

Before feature extraction, conversion of the CT scan intensities to Hounsfield Units (HU) was performed. The features can be divided in several groups. Intensity features were extracted using the histogram of all intensity values within the ROIs and included several first-order statistics such as the mean, standard deviation and kurtosis. These describe the distribution of Hounsfield units within the lesion. Shape features were extracted based only on the ROI, i.e. not using the image, and included shape descriptions such as the volume, compactness and circular variance. These describe the morphological properties of the lesion. Orientation features were used to describe the orientation of the ROI, i.e. not using the image. Lastly, texture features were extracted using Gabor filters, Laplacian of Gaussian filters, vessel (i.e. tubular structures) filters [9], the Gray Level Co-occurrence Matrix [7], the Gray Level Size Zone Matrix [7], the Gray Level Run Length Matrix [7], the Gray Level Dependence Matrix [7], the Neighbourhood Grey Tone Difference Matrix [7], Local Binary Patterns [10], and local phase filters [11, 12]. These features describe more complex patterns within the lesion, such as heterogeneity, occurrence of blob-like structures, and presence of line patterns.

Most of the texture features include parameters to be set for the extraction. Beforehand the values of the parameters that will result in features with the highest discriminative power for the classification at hand (i.e., dHGP versus rHGP) are not known. Including these parameters in the workflow optimization, see **Supplemental Materials B**, would lead to repeated computation of the features, resulting in a redundant decrease in computation time. Therefore, alternatively, these features are extracted at a range of parameters as is default in WORC. The hypothesis is that the features with high discriminative power will be selected by the feature selection methods and/or the machine learning methods as described in **Supplemental Materials B**. The parameters used are described in the caption of **Supplemental Table S2**.

The imaging data used in this study is multi-center, and therefore heterogeneous in terms of acquisition protocols. Especially the variations in slice thickness may cause feature values to be highly dependent on the acquisition protocol. Hence, extracting robust 3D features may be hampered by these variations, especially for low resolutions. The images were not resampled, as this would result in interpolation errors. To overcome this issue, all features were extracted per 2D axial slice and aggregated over all slices. Afterwards, several first-order statistics over the feature distributions were evaluated and used in the machine learning approach. As all images had the same unit (Hounsfield), no additional normalization was applied.

**Supplementary Materials B. Model optimization**

This supplementary material is similar to Vos et al. (2019) [1] and Timbergen et al. (2020) [2], but details relevant for the current study are highlighted.

The Workflow for Optimal Radiomics Classification (WORC) toolbox [3] makes use automated machine learning to create the optimal performing workflow from a variety of algorithms. Besides deciding whether to use an algorithm, most algorithms require hyperparameters, i.e., parameters that need to be set before the actual learning step, to be tuned to enhance the performance. WORC defines a workflow as a specific sequential combination of algorithms and their respective hyperparameters. In WORC, the radiomics workflow is split into the following components: image and segmentation preprocessing, feature extraction, feature and sample preprocessing, and machine learning. For each component, a collection of algorithms and their associated hyperparameters is included. Given this search space, WORC uses automated machine learning to find the optimal solution. The code to use WORC for creating the decision models in this specific study has been published open-source [6].

The workflows could be constructed from the following default search space in WORC, which components can only be combined in the order listed below:

1. Feature group selection: a group-wise search, in which specific groups of features (i.e., intensity, shape, and the subgroups of texture features as defined in **Supplementary Table S2**) are selected or deleted. To this end, each feature group had an on/off variable which is randomly activated or deactivated, which were all included as hyperparameters in the optimization.
2. Feature imputation: when a feature could not be computed, e.g. a lesion is too small for a specific feature to be extracted, a feature imputation algorithm was used to estimate replacement values for the missing values. Strategies for imputation included 1) the mean; 2) the median; 3) the mode; 4) a constant (default: zero); and 5) a nearest neighbor approach.
3. Feature selection: a variance threshold, in which features with a low variance (<0.01) are removed. This method was always used, as this serves as a feature sanity check with almost zero risk of removing relevant features.
4. Feature scaling was performed to make all features have the same scale, as otherwise the machine learning methods may focus only on those features with large values. This was done through z-scoring, i.e., subtracting the mean value followed by division by the standard deviation, for each individual feature. A robust version of z-scoring was used, in which outliers, i.e., values below the 5th percentile or above the 95th percentile, were excluded from computing the mean and variance.
5. Feature selection: optionally, the RELIEF method [13], which ranks the features according the differences between neighboring samples. Features with more differences between neighbors of different classes (i.e., dHGP versus rHGP) are considered higher in rank.
6. Feature selection: optionally, features are selected by training a machine learning model and selecting features that are regarded important by the model. Hence the used model should be able to give the features an importance weight. Included model choices are LASSO, logistic regression, and a random forest.
7. Dimensionality reduction: optionally, principal component analysis (PCA) is used, in which either only those linear combinations of features were kept which explained 95% of the variance in the features or a limited number of components (between 10 – 50).
8. Feature selection: optionally, individual features were selected through univariate testing. To this end, for each feature, a Mann-Whitney U test was performed to test for significant differences in distribution between the labels (i.e., dHGP versus rHGP). Afterwards, only features with a p-value above a certain threshold were selected.
9. Resampling: optionally, a resampling strategy could be used, which was used to overcome class imbalances and reduce overfitting on specific training samples. Various methods from the imbalanced-learn toolbox [14] could be used: random over-sampling, random under-sampling, near-miss resampling, the neighborhood cleaning rule, ADASYN, and SMOTE (regular, borderline, Tomek and the edited nearest neighbors variant).
10. Machine learning: lastly, a machine learning methods was used to determine a decision rule to distinguish the classes. Methods included were; 1) logistic regression; 2) support vector machines; 3) random forests; 4) naive Bayes; 5) linear discriminant analysis; 6) quadratic discriminant analysis; 7) AdaBoost [15]; and 8) extreme gradient boosting [16].

The performance of WORC was evaluated in this study through a 100x random-split cross-validation [17, 18], in each iteration splitting the data in 80% for training and 20% for testing. In each cross-validation iteration, all optimization was performed on the training set in order to prevent overfitting on the test set. To prevent overfitting on the *training* dataset, a 5x random-split stratified cross-validation was performed within the training dataset as well, using 85% for model training and 15% for model validation, see **Supplementary Fig. S2**.

WORC states the radiomics workflow as a combined algorithm selection and hyperparameter optimization problem (CASH), as algorithm selection and hyperparameter optimization are often not independent [19]. In each training-test cross-validation iteration, CASH optimization is performed within the training dataset by testing thousand pseudo-randomly generated radiomics workflows from the above search space. These are trained on the five training sets in the 5x random-split training-validation cross-validation, and ranked according to their mean performance on the five validation datasets. As performance metric, the weighted F1-score is used, which is the weighted harmonic average of the precision and recall.

Using only the single workflow that on average performs best on the validation sets may result in poor generalization due to overfitting on the validation sets. Hence, an ensemble was constructed by combining the workflows that perform best on the validation sets [20] . Ensembling was done using the default of WORC by averaging the posteriors of the 100 best workflows.

The following pseudo code illustrates the algorithm of WORC:

- **For** each 100x random-split training-test cross-validation iteration:
  - **Do:** Construct the training dataset by randomly selecting 80% of the patients.
  - **Do:** On this training dataset, define 5x random-split cross-validation splits, selecting in each iteration 85% of the patients for training and 15% for validation.
  - **Do:** Pseudo-randomly sample 1,000 workflows from the search space.
  - **For** each of the 1,000 sampled workflows:
    - **Do:** Train the workflow on the five training datasets in the 5x random-split cross-validation.
    - **Do:** Compute the mean weighted F1-score on the corresponding five validation datasets in the 5x random-split cross-validation.
  - **Do**: Rank the 1,000 workflows, retrain the best 100 workflows on the full training set, and combine them into an ensemble model.
  - **Do:** Evaluate the ensemble model on the test dataset, i.e., the remaining 20% of the patients that were not included in the training dataset.

**Supplementary References**

1. Vos M, et al. (2019) Radiomics approach to distinguish between well differentiated liposarcomas and lipomas on MRI. British Journal of Surgery 106 13): 1800-9. DOI: 10.1002/bjs.11410

2. Timbergen MJM, et al. (2020) Differential diagnosis and mutation stratification of desmoid-type fibromatosis on MRI using radiomics. European Journal of Radiology 131: 109266. DOI: 10.1016/j.ejrad.2020.109266

3. Starmans MPA, et al. (2018) Workflow for Optimal Radiomics Classification (WORC). <https://github.com/MStarmans91/WORC>. Accessed: 25-02-2021. DOI: 10.5281/zenodo.3840534

4. van der Voort SR, Starmans MPA (2018) Predict: a Radiomics Extensive Digital Interchangable Classification Toolkit (PREDICT). <https://github.com/Svdvoort/PREDICTFastr>. Accessed: 25-02-2021. DOI: 10.5281/zenodo.3854839

5. Van Griethuysen JJ, et al. (2017) Computational radiomics system to decode the radiographic phenotype. Cancer research 77 21): e104-e7. DOI: 10.1158/0008-5472.CAN-17-0339

6. Starmans MPA (2021) CLMRadiomics. <https://github.com/MStarmans91/CLMRadiomics>. Accessed: 20-07-2021. DOI: 10.5281/zenodo.4392829

7. Zwanenburg A, et al. (2020) The Image Biomarker Standardization Initiative: Standardized Quantitative Radiomics for High-Throughput Image-based Phenotyping. Radiology 295: 191145. DOI: 10.1148/radiol.2020191145

8. Starmans MPA (2018) Workflow for Optimal Radiomics Classification (WORC) Documentation. <https://worc.readthedocs.io>. Accessed: 25-02-2021. DOI: 10.5281/zenodo.3840534

9. Frangi AF, et al. (1998) Multiscale vessel enhancement filtering. In: Wells WM, Colchester A, Delp S (eds) Medical Image Computing and Computer-Assisted Intervention — MICCAI'98. Springer Berlin Heidelberg, p. 130-7.DOI: 10.1007/BFb0056195

10. Ojala T, Pietikainen M, Maenpaa T (2002) Multiresolution gray-scale and rotation invariant texture classification with local binary patterns. IEEE Transactions on Pattern Analysis and Machine Intelligence 24 7): 971-87. DOI: 10.1109/TPAMI.2002.1017623

11. Kovesi P (2003) Phase congruency detects corners and edges. The Australian pattern recognition society conference: DICTA.

12. Kovesi P (1997) Symmetry and asymmetry from local phase. Tenth Australian joint conference on artificial intelligence, vol 190. Citeseer, p. 2–4

13. Urbanowicz RJ, et al. (2018) Benchmarking relief-based feature selection methods for bioinformatics data mining. Journal of Biomedical Informatics 85: 168-88. DOI: 10.1016/j.jbi.2018.07.015

14. Lemaitre G, Nogueira F, Aridas CK (2017) Imbalanced-learn: A Python Toolbox to Tackle the Curse of Imbalanced Datasets in Machine Learning. Journal of Machine Learning Research 18

15. Freund Y, Schapire RE (1997) A Decision-Theoretic Generalization of On-Line Learning and an Application to Boosting. Journal of Computer and System Sciences 55 1): 119-39. DOI: 10.1006/jcss.1997.1504

16. Chen T, et al. (2015) Xgboost: extreme gradient boosting. R package version 04-2: 1-4.

17. Picard RR, Cook RD (1984) Cross-Validation of Regression Models. Journal of the American Statistical Association 79 387): 575-83. DOI: 10.1080/01621459.1984.10478083

18. Nadeau C, Bengio Y (2003) Inference for the Generalization Error. Machine Learning 52 3): 239-81. DOI: 10.1023/A:1024068626366

19. Thornton C, et al. (2013) Auto-WEKA: Combined selection and hyperparameter optimization of classification algorithms. p. 847-55 DOI: 10.1145/2487575.2487629

20. Zhang C, Ma Y (2012) Ensemble Machine Learning. Springer, New York.DOI: 10.1007/978-1-4419-9326-7
