# Supplementary material for: Distinguishing pure histopathological growth patterns of colorectal liver metastases on CT using deep learning and radiomics: a pilot study
Source: Clin Exp Metastasis. 2021 Sep 17;38(5):483–94. doi: 10.1007/s10585-021-10119-6 (PMC8510954; doi:10.1007/s10585-021-10119-6)
Supplement: Supplementary file 2 — Supplementary file1 (Table S1): Segmentation agreement expressed in Dice Similarity Coefficient (DSC) (mean (standard deviation)) between the observers and the convolutional neural network (CNN) (STUD (1st and 2nd time), PhD, RAD, CNN). The average of the mean and standard deviation of the DSC for each observer are stated in the bottom row (DOCX 13 kb) [file 10585_2021_10119_MOESM2_ESM.docx]

| **Observer** | **STUD1** | **STUD2** | **PhD** | **RAD** | **CNN** |
| --- | --- | --- | --- | --- | --- |
| **STUD1** | - | 0.80 (0.15) | 0.73 (0.14) | 0.60 (0.18) | 0.65 (0.26) |
| **STUD2** | 0.80 (0.15) | - | 0.77 (0.13) | 0.63 (0.18) | 0.66 (0.27) |
| **PhD** | 0.73 (0.14) | 0.77 (0.13) | - | 0.69 (0.16) | 0.63 (0.25) |
| **RAD** | 0.60 (0.18) | 0.63 (0.18) | 0.69 (0.16) | - | 0.58 (0.27) |
| **CNN** | 0.65 (0.26) | 0.66 (0.27) | 0.63 (0.25) | 0.58 (0.27) | - |
| **Average** | 0.70 (0.18) | 0.72 (0.18) | 0.71 (0.17) | 0.63 (0.20) | 0.63 (0.26) |
