# Supplementary material for: Distinguishing pure histopathological growth patterns of colorectal liver metastases on CT using deep learning and radiomics: a pilot study
Source: Clin Exp Metastasis. 2021 Sep 17;38(5):483–94. doi: 10.1007/s10585-021-10119-6 (PMC8510954; doi:10.1007/s10585-021-10119-6)
Supplement: Supplementary file 3 — Supplementary file6 (Table S2): Overview of the 564 features used in this study. GLCM features were calculated in four different directions (0, 45, 90, 135 degrees) using 16 gray levels and pixel distances of 1 and 3. LBP features were calculated using the following three parameter combinations: 1 pixel radius and 8 neighbours, 2 pixel radius and 12 neighbours, and 3 pixel radius and 16 neighbours. Gabor features were calculated using three different frequencies (0.05, 0.2, 0.5) and four different angles (0, 45, 90, 135 degrees). LoG features were calculated using three different widths of the Gaussian (1, 5 and 10 pixels). Vessel features were calculated using the full mask, the edge, and the inner region. Local phase features were calculated on the monogenic phase, phase congruency and phase symmetry (DOCX 17 kb) [file 10585_2021_10119_MOESM3_ESM.docx]

| Histogram  (13 features) | LoG  (13*3=39 features) | | Vessel  (12*3=39 features) | GLCM (MS)  (6*3*4*2=144 features) | | Gabor  (13*4*3=156 features) | NGTDM  (5 features) | LBP  (13*3=39 features) |  |
| --- | --- | --- | --- | --- | --- | --- | --- | --- | --- |
| min  max  mean  median  std  skewness  kurtosis  peak  peak position  range  energy  quartile range  entropy | min  max  mean  median  std  skewness  kurtosis  peak  peak position  range  energy  quartile  entropy | | min  max  mean  median  std  skewness  kurtosis  peak  peak position  range  energy  quartile  entropy | contrast (normal, MS mean + std)  dissimilarity (normal, MS mean + std)  homogeneity(normal, MS mean + std)  angular second moment (ASM) (normal, MS mean + std)  energy (normal, MS mean + std)  correlation (normal, MS mean + std) | | min  max  mean  median  std  skewness  kurtosis  peak  peak position  range  energy  quartile range  entropy | busyness  coarseness  complexity  contrast  strength | min  max  mean  median  std  skewness  kurtosis  peak  peak position  range  energy  quartile range  entropy |  |
| GLSZM  (16 features) | | **GLRM**  **(16 features)** | | | **GLDM**  **(14 features)** | **Shape**  **(35 features)** | **Orientation**  **(9 features)** | **Local phase**  **(13*3=39 features)** |  |
| Gray Level Non Uniformity  Gray Level Non Uniformity Normalized  Gray Level Variance  High Gray Level Zone Emphasis  Large Area Emphasis  Large Area High Gray Level Emphasis  Large Area Low Gray Level Emphasis  Low Gray Level Zone Emphasis  SizeZoneNonUniformity  SizeZoneNonUniformityNormalized  SmallAreaEmphasis  SmallAreaHighGrayLevelEmphasis  SmallAreaLowGrayLevelEmphasis  ZoneEntropy  ZonePercentage  ZoneVariance | | Gray Level Non Uniformity  Gray Level Non Uniformity Normalized  Gray Level Variance  High Gray Level Run Emphasis  Long Run Emphasis  Long Run High Gray Level Emphasis  Long Run Low Gray Level Emphasis  Low Gray Level Run Emphasis  RunEntropy  RunLengthNonUniformity  RunLengthNonUniformityNormalized  RunPercentage  RunVariance  ShortRunEmphasis  ShortRunHighGrayLevelEmphasis  ShortRunLowGrayLevelEmphasis | | | Dependence Entropy  Dependence Non-Uniformity  Dependence Non-Uniformity Normalized  Dependence Variance  Gray Level Non-Uniformity  Gray Level Variance  High Gray Level Emphasis  Large Dependence Emphasis  Large Dependence High Gray Level Emphasis  Large Dependence Low Gray Level Emphasis  Low Gray Level Emphasis  Small Dependence Emphasis  Small Dependence High Gray Level Emphasis  Small Dependence Low Gray Level Emphasis | compactness (mean + std)  radial distance (mean + std)  roughness (mean + std)  convexity (mean + std)  circular variance (mean + std)  principal axes ratio (mean + std)  elliptic variance (mean + std)  solidity (mean + std)  area (mean, std, min + max  volume (total, mesh, volume)  elongation  flatness  least axis length  major axis length  minor axis length  maximum diameter 3D  maximum diameter 2D (rows, columns, slices)  sphericity  surface area  surface volume ratio | theta_x  theta_y  theta_z  COM index x  COM index y  COM index z  COM x  COM y  COM z | min  max  mean  median  std  skewness  kurtosis  peak  peak position  range  energy  quartile  entropy |  |
| *Abbreviations: COM: center of mass; GLCM: gray level co-occurrence matrix; MS: multi slice; NGTDM: neighborhood gray tone difference matrix; GLSZM: gray level size zone matrix; GLRLM: gray level run length matrix; LBP: local binary patterns; LoG: Laplacian of Gaussian; std: standard deviation. | | | | | | | |  |  |
