# Supplementary figures and images for: Distinguishing pure histopathological growth patterns of colorectal liver metastases on CT using deep learning and radiomics: a pilot study
Source: Clin Exp Metastasis. 2021 Sep 17;38(5):483–94. doi: 10.1007/s10585-021-10119-6 (PMC8510954; doi:10.1007/s10585-021-10119-6)

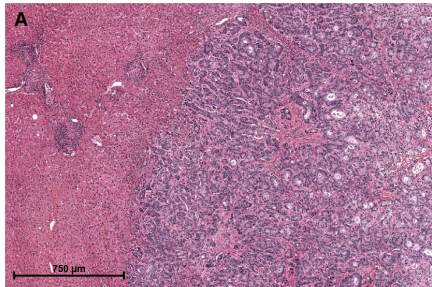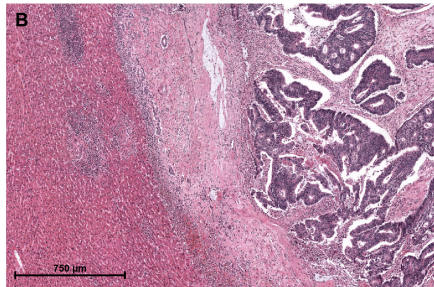

Supplement: Supplementary file 4 — Supplementary file2 (Figure S1): Replacement type (A) and desmoplastic type (B) histopathological growth pattern on hematoxylin and eosin stained tissue sections (PDF 4812 kb) [file 10585_2021_10119_MOESM4_ESM.pdf]

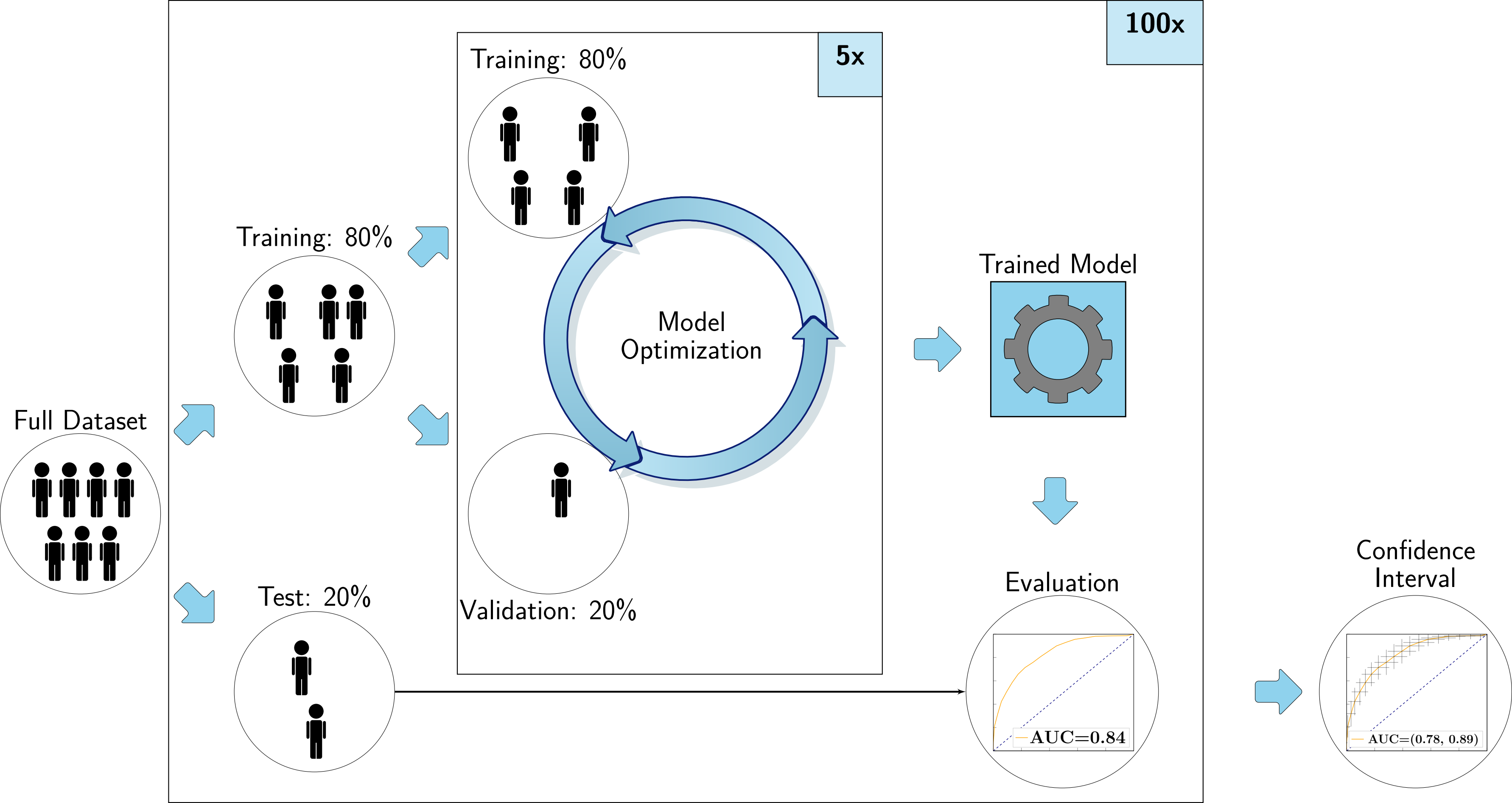

Supplement: Supplementary file 5 — Supplementary file3 (Figure S2): Visualization of the 100x random split cross-validation, including a second cross-validation within the training set for model optimization. The test dataset is only used for evaluation of the trained model (PDF 61 kb) [file 10585_2021_10119_MOESM5_ESM.pdf]

a.

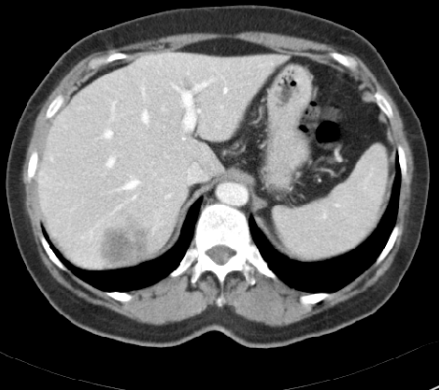

b.

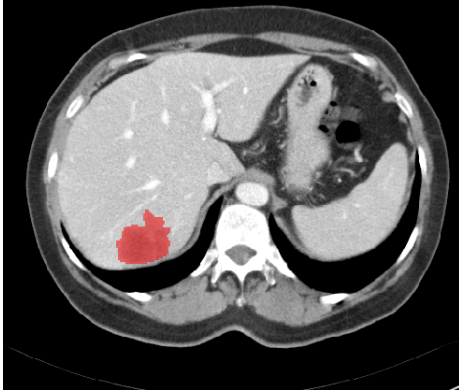

c.

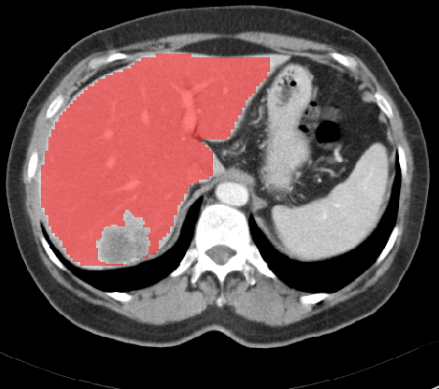

d.

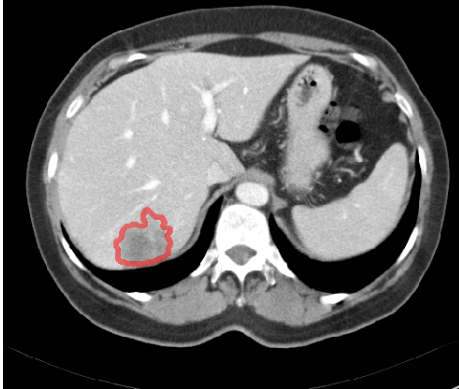

Supplement: Supplementary file 6 — Supplementary file4 (Figure S3): Examples of segmentations of various regions of interest on a single axial slice of CT-scans. A: CT-scan without segmentation; B: lesion; C: normal liver parenchyma; and D: ring on the border between the lesion and normal liver parenchyma (PDF 655 kb) [file 10585_2021_10119_MOESM6_ESM.pdf]
